# HER2 expression and pathway status in male breast cancer patients: results of an integrated analysis among 6,150 patients

**DOI:** 10.1038/s41598-025-86556-0

**Published:** 2025-01-27

**Authors:** Boqiang Lyu, Shidi Zhao, Hui Wang, Shouping Gong, Biyuan Wang

**Affiliations:** 1 Department of Neurosurgery, the Second Affiliated Hospital of Xi’an Jiaotong University, Xi’an, 710014 Shaanxi Province China; 2 Department of Medical Oncology, The First Affiliated Hospital of Xi’an Jiaotong University, Xi’an, 710061 Shaanxi China; 3 Xi’an Medical University, Xi’an, China; 4 Department of Gastroenterology, the Second Affiliated Hospital of Xi’an Jiaotong University, Xi’an, 710014 Shaanxi Province China; 5 The Second Affiliated Hospital of Xi’an Jiaotong University, Xi’an, 710014 Shaanxi Province China

**Keywords:** Human epidermal growth factor receptor, Immunohistochemistry, Fluorescence in situ hybridization, Cancer, Oncology

## Abstract

The role of human epidermal growth factor 2 (HER2) in male breast cancer (MBC) is poorly defined. A comprehensive description of HER2 status was conducted. A total of 6,015 MBC patients from 45 studies and 135 MBC patients with sequencing data were identified. HER2 positive rates and hazard ratios (HR) for overall survival (OS) were combined using Metaprop. The prevalence of HER2 + MBC was 10.0% (95% CI: 8.0-13.0%). Subgroup analyses showed that 7% (95% CI: 2.0-14.0%) had HER2 + protein overexpression. 10% of MBC patients had HER2 + overexpression and/or gene amplification. Asian MBC patients had the highest HER2 + incidence of 17% (95% CI: 12.0-22.0%). The prevalence of HER2 positive MBC fluctuated widely from 2001 to 2015 and then stabilized at 10%. HER2 positivity was significantly correlated with worse OS than negative ones (HR = 1.92, 1.47–2.51). The proportion of HER2 + MBC was inconsistent with the results for the intrinsic HER2-enriched subtype. Altered genes in HER2 + MBC, such as ERBB2, AGO2, RECQL4, and CLTC, were not detected in HER2-MBC. Genomic analysis revealed differences between the patients with HER2 + MBC and those with HER2 + FBC. The percentage of HER2 + MBC was slightly lower than that of women. Multiple approaches may be needed to jointly assess HER2 status in MBC.

**Supplementary Information:**

The online version contains supplementary material available at 10.1038/s41598-025-86556-0.

## Introduction

About 1% of all patients with breast cancer are diagnosed with men^1,2^. The incidence of male breast cancer (MBC) is related to genetic disorders (e.g., Klinefelter syndrome) and family history (e.g., breast/ovarian cancer). Approximately 10% of MBC patients have a BRCA2 mutation, while BRCA1 mutations are less common. Other genes associated with hereditary MBC have been identified gradually, including those related to DNA damage response (DDR), such as ATM, PALB2, CHEK2 and BRIP1^3–6^. Additionally, hormonal imbalances caused by obesity, hepatic insufficiency and exogenous estrogens increase the risk of MBC. According to global data, MBC are more likely to be Black^7^and always older than female breast cancer patients, more likely to be ductal hormone receptor positive^1,8^. The incidence^2^and mortality^7,9,10^ of MBC have increased, revealing the necessity of exploring and implementing appropriate treatment options.

Due to the rarity of MBC, it is difficult to conduct clinical trials, making it challenging to meet the clinical management. Thus, treatment strategies are usually extrapolated from female breast cancer (FBC). Observational and retrospective studies from medical centers worldwide are important, as well as genomic data. Since MBC is mainly estrogen receptor (ER)-positive and progesterone receptor (PR)-positive, some investigators believe that it is less aggressive than FBC and more similar to post-menopausal FBC^11^.But other studies have observed a larger size at diagnosis and a worse overall survival (OS) rate in MBC than those in FBC patients^10,12,13^. According to the results of the EORTC 10,085/TBCRC/BIG/NABCG International Male Breast Cancer Program, the rates of adjuvant endocrine therapy or radiotherapy/breast-conserving surgery are lower than those of FBC^7,8^. The poor prognosis of MBC patients has raised concerns among researchers.

The intrinsic differences between MBC and FBC are interesting regarding their genomic characteristics and treatment modes. Previous studies on the 4-gene traditional classifier and molecular classifiers based on the multigene signature of MBC have provided inconsistent results^12,14–16^. MBC is always recognized as the luminal type. The percentage of human epidermal growth factor receptor-2 (HER2)-positive type has been reported to range from almost zero to approximately 15% or close to 30%^13,15,17,18^. The differences between these studies make things complicated and confusing. Different detection methods for HER2 amplification and expression may partly explain this discrepancy. HER2 protein overexpression is generally evaluated by immunohistochemistry (IHC) and fluorescence in situ hybridization (FISH) for HER2 gene amplification. Bright-field in situ hybridization (BISH) methods, including chromogenic in situ hybridization (CISH) and silver in situ hybridization (SISH), have been developed to overcome some limitations of FISH. The criteria for HER2 positivity have been adjusted several times in the past two decades, such as modifications to the cutoff values of IHC and FISH methods. The prognostic role of HER2 remains poorly understood.

Our aim was to clarify HER2 status and its role in MBC by analyzing HER2 + MBC data from 45 studies and 135 samples from Memorial Sloan-Kettering Cancer Center (MSK), The Cancer Genome Atlas (TCGA), and the Gene Expression Omnibus (GEO) databases. In addition, we aimed to identify the activation status of HER2 signaling to explore potential MBC patients who might benefit from anti-HER2 therapy. Genomic features were extracted to identify other therapeutic targets in HER2 + MBC.

## Materials and methods

### Data acquisition

We performed a search on Embase, Pubmed, Scopus, web of science and the Cochrane Library, as well as cnki, Wanfangdata. The search strategy was: “human epidermal growth factor 2” or “HER2” or “Her2/neu” or “c-erb2” or “Erbb2” combined with “male breast cancer.” Two reviewers (WH and ZSD) independently screened the articles and identified studies that met the following inclusion criteria: articles reporting the status of HER2 expression among MBC patients and all studies published after 1998, the year trastuzumab received FDA approval. Relevant studies in the reference lists of these articles were also searched. Conference abstracts, case reports, letters, reviews, and studies without primary data were excluded. Thus, we included all articles that reported HER2 status. To assess the quality of these studies, two researchers (WBY and ZSD) independently extracted and evaluated the data. The JBI Critical Appraisal Checklist was used to evaluate the quality of all the studies^19^. There were ten questions in the JBI checklist that needed to be answered regarding completeness, accuracy, and risk of bias. The studies included in our analysis had JBI scores greater than 5 to ensure quality. Based on the validation results (Supplementary Table S1), information including race, methods including IHC, ISH (e.g.,: FISH, SISH and CISH), and the number of patients with valid tissue detection, as well as other detailed information, were collected for further analysis^20^. The prevalence in each publication for conducting meta-analysis was determined based on the positive standards at that time.

### Patient selection

We searched the open genomic database for Cancer Genomics and extracted data of 23 MBC tumor tissues using cBioPortal (http://cbioportal.org). There were 23 MBC patients from TCGA and MSK, and their clinicopathological features, molecular characteristics, transcriptional gene profiles, and copy number variation (CNV)/gene mutation information were collected^21^. Similarly, 199 HER2 + FBC patients from MSK and 177 HER2 + FBC patients from TCGA were identified. Their genomic data were collected for comparative analysis. To better understand the molecular features of MBC, gene expression profiles of breast cancer tissues (GSE31259, *n* = 66; GSE104730, *n* = 46) were selected from the GEO database.

### Data analysis

We used the metaprop method to calculate the incidence of HER2 + MBC along with the 95% confidence interval (CI) using R Studio software. A comprehensive meta-analysis program version 3 (Biostat, Englewood, NJ, USA) was used to perform a cumulative analysis of all studies. The reported hazard ratios (HRs) and 95% CI from the univariate analysis between OS and HER2 expression were also analyzed using R studio. When the HR was not presented in the article, the Engauge Digitizer was used to calculate survival data from the Kaplan-Meier curves and to determine HRs and 95% CIs. When the heterogeneity among studies was high (I^2^ > 50%), the random-effects model was used for the meta-analysis rather than the fixed-effects model. A funnel chart was used to assess publication bias. *P* < 0.05 was set as the significance level.

### Identification of HER2 signaling status in breast cancer

The Genefu” R/Bioconductor package was used to generate PAM50 subtypes based on 50 gene expression signatures in breast cancer^22^. “HER2-enriched” subtype means the cancer that is driven by HER2 pathway. The mRNA expression levels of genes within “Biocarta her2 pathway” gene-set among TCGA MBC patients were clustered and shown in the heatmap.

### Gene set enrichment analysis

Mutated genes frequently observed in HER2 + MBC patients were uploaded online to conduct protein-protein interaction (PPI) networks by using the “string” database^23^. The Kyoto Encyclopedia of Genes and Genomes (KEGG) pathway enrichment analysis was performed using the “STRING” database. The threshold for significant pathways was set at a false discovery rate (FDR) < 0.05.

### Drug-gene interaction analysis

After the list of mutated genes was obtained, chord and Sankey diagrams were generated using the SRPlot^24^. The Drug-Gene Interaction Database (DGIdb 4.0) is a tool for annotating and verifying potential drugs for HER2 + MBC^25^.

## Results

### Systematic review of HER2 + MBC

**Fig. 1:**
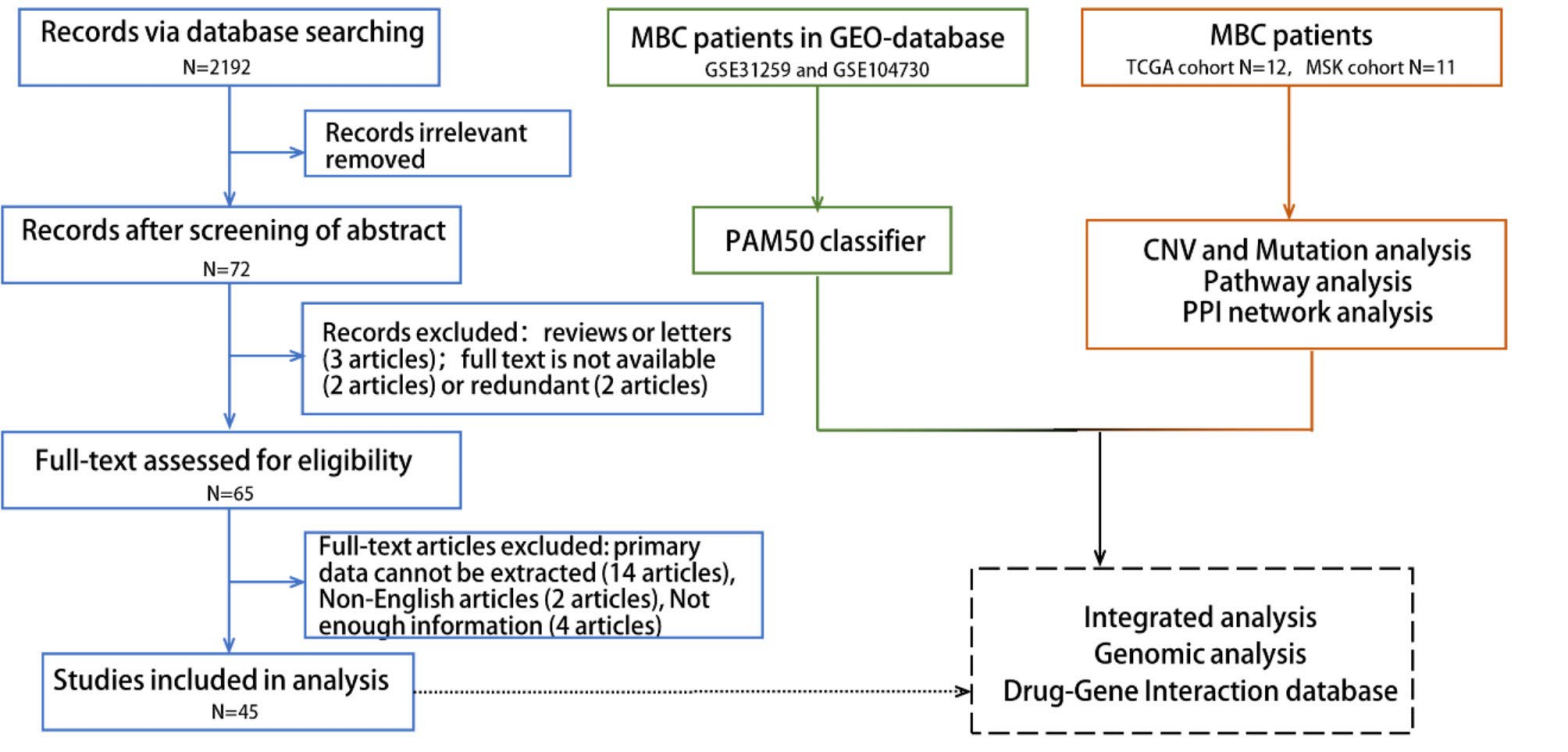
Study flowchart. Selection of publications from database and genomic database involved, including mRNA level, pathway activation and gene alteration of MBC.

A total of 6,015 patients from 45 studies fulfilled the inclusion criteria after screening (Fig. 1). The characteristics of these studies were summarized in Table 1.

The 45 retrospective studies were of high quality (quality scores of 6–10)^8,13,15–18,26–64^. The proportion of HER2 positive cases in the 45 studies varied from 0 to 30.0%. Thirty-nine studies mentioned the methods used to assess HER2 expression. The cutoff value for the percentage of tumor cells expressing HER2 protein strongly on the membrane, as well as the cutoff value for the Her2/CEP17 ratio, has been adjusted for a period over 20 years, as shown in the table.

**Table 1:** Characteristics and basic information of 45 involved articles.

ID^REF^	Year	Country	Test Method	*N*.*	IHC Cut-off Value (% of cells)	Her2/CEP17 Ratio Cut-off Value	Informations of Antibody
1^22^	2001	U.S.A.	IHC+FISH	55	10	2	Ventana Medical Systems, CB11
2^23^	2002	Italy	IHC	27	10	NA	Biogenex, CB11
3^24^	2003	Canada	IHC	59	NA	NA	DAKO
4^25^	2004	Germany	IHC+FISH	99	10	2	DAKO
5^26^	2006	Portugal	IHC+FISH	50	10	2	Ventana Medical Systems
6^27^	2007	Canada	IHC	42	10	NA	DAKO Corp
7^28^	2009	India	IHC	20	10	NA	Novocastra, CB11
8^29^	2009	U.S.A.	IHC+FISH	42	10	2	NeoMarkers
9^30^	2011	Germany	IHC+FISH	77	NA	NA	NA
10^31^	2012	Turkey	IHC+FISH	77	NA	NA	NA
11^32^	2012	Lebanon	IHC+FISH	22	NA	NA	DAKO Corp
12^14^	2012	Netherlands	IHC+FISH	130	NA	NA	NeoMarkers, SP3
13^11^	2012	UK.	IHC	251	NA	NA	NA
14^33^	2012	Australia	SISH	55	NA	NA	NA
15^34^	2013	Italy	NA	105	30	2.2	NA
16^35^	2013	Sweden	IHC+SISH	197	NA	2.2	Ventana Medical Systems,4B5
17^13^	2013	U.S.A.	NA	606	NA	NA	NA
18^36^	2013	China	IHC+FISH	64	NA	NA	NA
19^37^	2014	Serbia	IHC+FISH	84	NA	2.3	NA
20^38^	2015	India	IHC+FISH	53	NA	2	Thermo
21^39^	2015	Italy	IHC+FISH	85	30	2.2	DAKO
22^40^	2015	Netherlands	IHC+FISH	69	NA	NA	Roche,4B5
23^41^	2016	Korea	IHC	148	NA	NA	NA
24^42^	2016	Iran	IHC+FISH	17	NA	NA	NA
25^43^	2016	Italy	IHC+FISH	41	NA	NA	NA
26^44^	2016	Portugal	IHC+FISH	111	10	2	NA
27^45^	2016	U.S.A.	NA	59	10	2	NA
28^46^	2017	U.S.A.	IHC+FISH	60	10	2	Ventana Medical Systems CB11/4B5
29^47^	2017	UK.	IHC+FISH	297	10	2	NA
30^48^	2017	Tunis	IHC+CISH	130	NA	NA	Novocastra, CB11
31^49^	2018	Turkey	IHC+SISH	50	NA	NA	NA
32^12^	2018	Spain	IHC+CISH	67	10	2	DAKO Corp
33^50^	2018	Netherlands	IHC	164	10	NA	NeoMarkerS
34^3^	2018	UK, U.S.A., Netherlands	IHC+FISH	1044	10	2	Ventana Medical Systems,4B5
35^51^	2019	China	NA	57	NA	NA	NA
36^52^	2019	India	IHC+FISH	42	10	2	Ventana Medical Systems,4B5
37^53^	2019	Italy	NA	323	NA	NA	NA
38^9^	2020	China	IHC+FISH	63	NA	NA	NA
39^54^	2020	Turkey	IHC+ISH	27	NA	NA	DAKO Corp
40^55^	2020	Romania	IHC+FISH	204	NA	NA	Novocastra, CB11
41^56^	2020	Denmark	IHC+SISH	373	NA	NA	NA
42^57^	2021	China	IHC	90	NA	NA	Mbiotech
43^58^	2021	Turkey	IHC+FISH	41	NA	NA	NA
44^59^	2021	Poland	NA	125	NA	NA	NA
45^60^	2022	Korea	IHC+SISH	213	NA	NA	NA

Notes: ^a^ Patients whose FISH status of HER2 was not tested were excluded. Abbreviations: NA, not available.

**Fig. 2:**
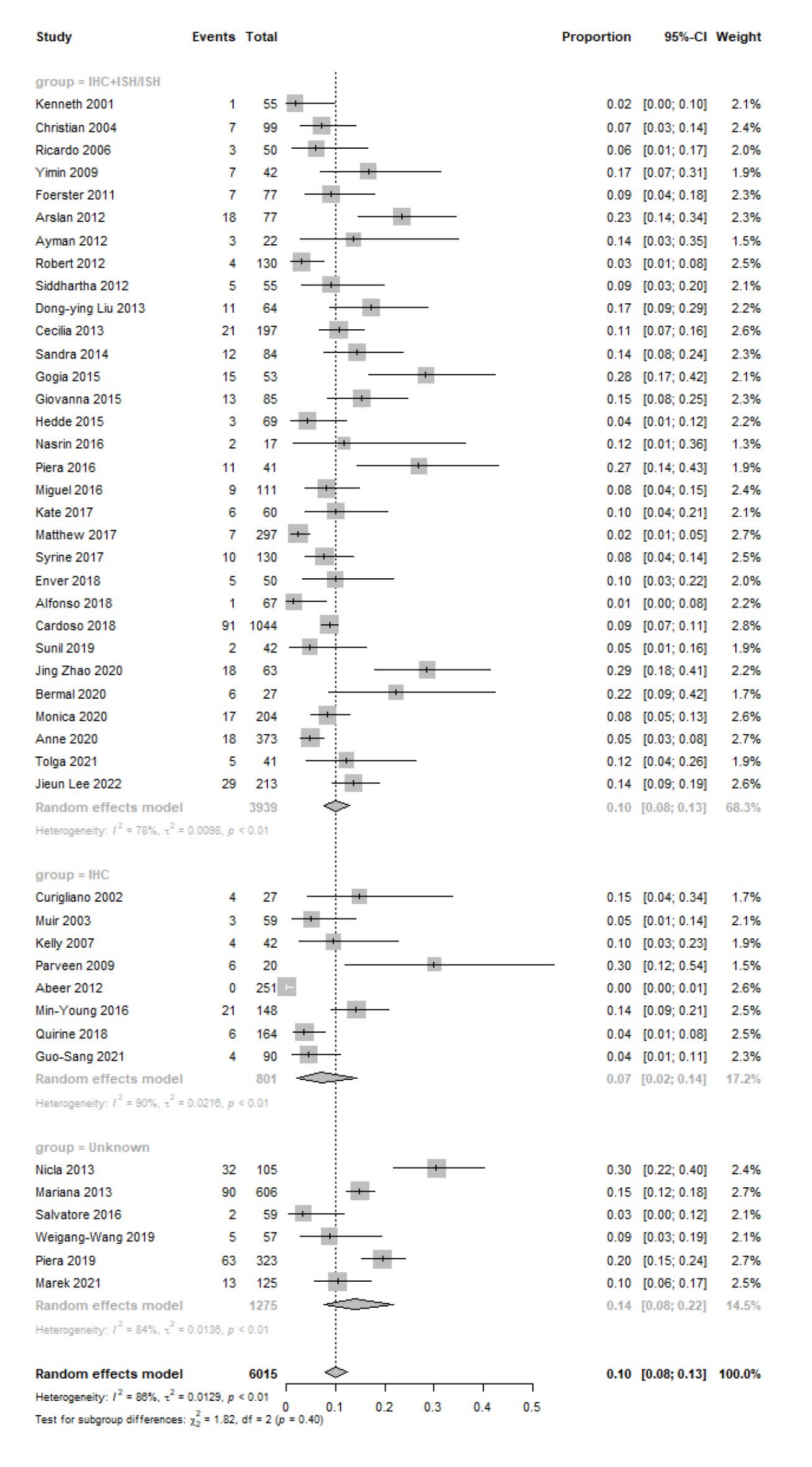
HER2 + event rates in MBC studies grouped by test methods.

The data were grouped by method into three subtypes: ISH or ISH + IHC (Group1, *n* = 31), only IHC (Group 2, *n* = 8) and test information not mentioned (Group 3, *n*= 6). The pooled event rate of HER2 status among the 45 studies was 10.0% (95% CI: 8.0–13.0%), with statistically significant heterogeneity (I^2^ = 86%, *P* < 0.01) (Fig. 2). There was no significant result from the Egger test in the funnel plot (*P* = 0.24) (Supplementary Fig. 1).

The meta-analysis revealed that Group 2 exhibited the lowest prevalence of HER2 positivity (7.0%, 95% CI: 2.0–14.0%). Heterogeneity was also found in Group 2 (I^2^ = 90%, *P* < 0.01). In studies evaluating HER2 amplification, namely Group 1, the rate was 10.0% (95% CI: 8.0–13.0%). The studies in group 1 showed significant heterogeneity (I^2^ = 78%, *P* < 0.01). For the remaining studies with unspecified test methods, the pooled HER2 overexpression rate was 14.0% (95% CI: 8.0–22.0%) with significant heterogeneity (I^2^ = 84%, *P* < 0.01). Among the three subgroups, only group 1 had publication bias according to the funnel plot (*p* = 0.04). The Egger test results for the other two groups were not statistically significant (Group2: p-value = 0.07 and Group3: p-value = 0.63).

**Fig. 3:**
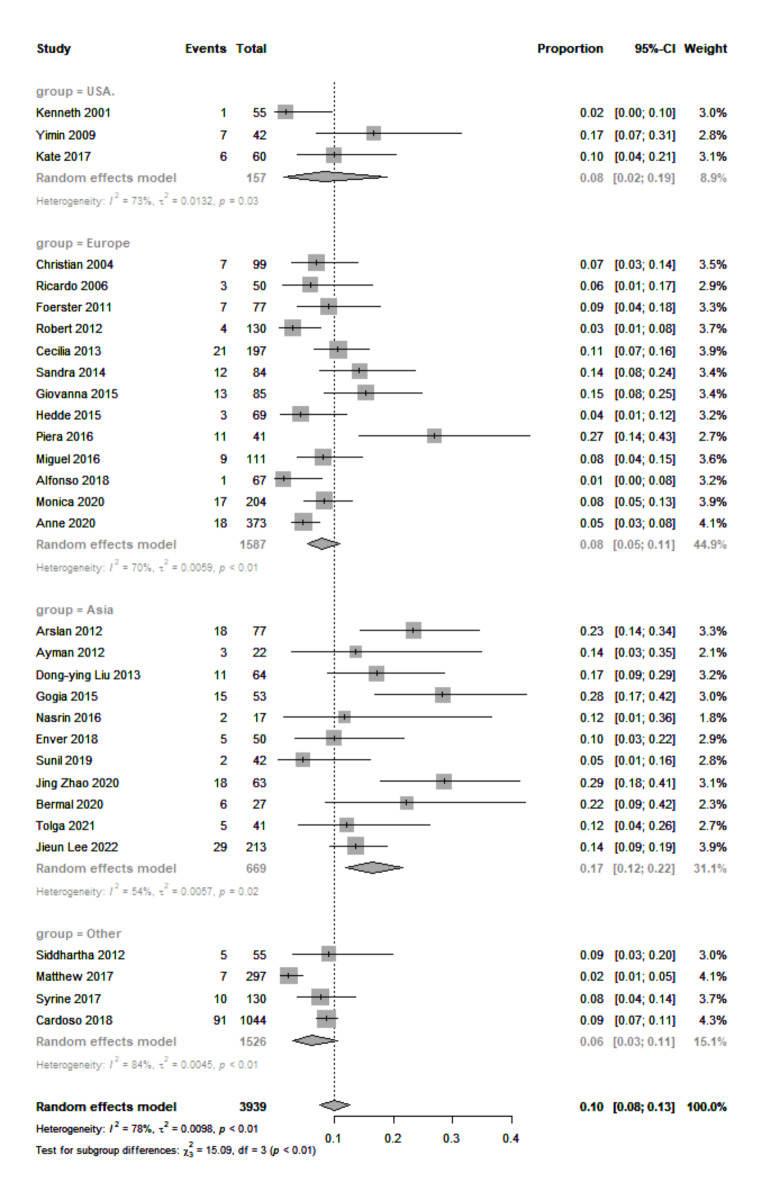
HER2 + event rates in MBC studies grouped by country.

Furthermore, we demonstrated the regional impact of HER2 + status in Group 1 (Fig. 3). Asia had the highest positivity rate at 17% (95% CI: 12.0–22.0%). The heterogeneity was lower than previously observed (I^2^ = 54%, *P* = 0.02). Europe and the United States had similar HER2 + event rates of 8.0% (95% CI: 5.0–11.0%) and 8.0% (95% CI: 2.0–19.0%), respectively.

**Fig. 4:**
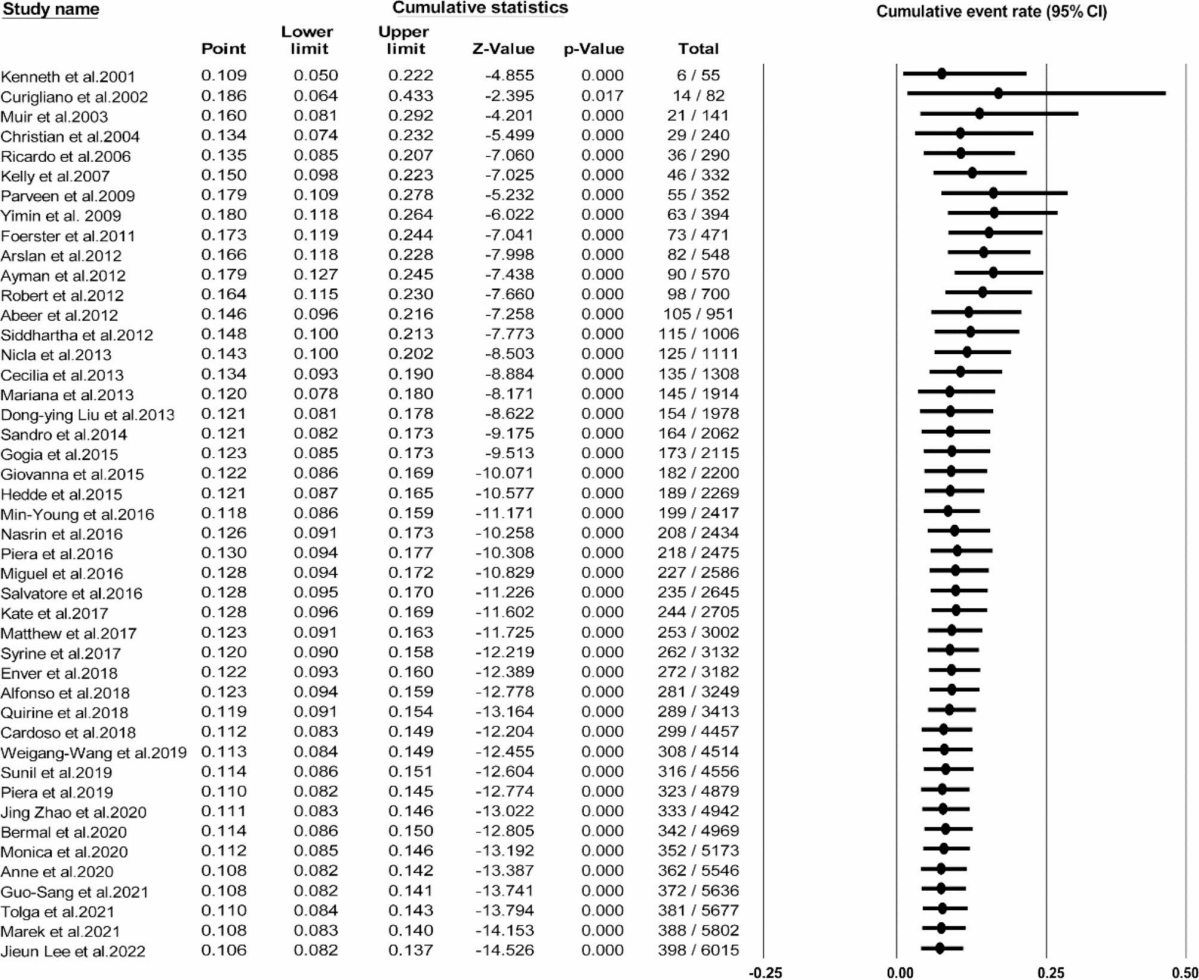
Forest plot of cumulative prevalence and 95% CI of HER2 + MBC from 2001 to 2022.

As shown in Fig. 4, the cumulative prevalence of HER2 + MBC exhibited significant variations before 2014. There is a slight variation, ranging from 10.6 to 12.8% between 2016 and 2022.

**Fig. 5:**
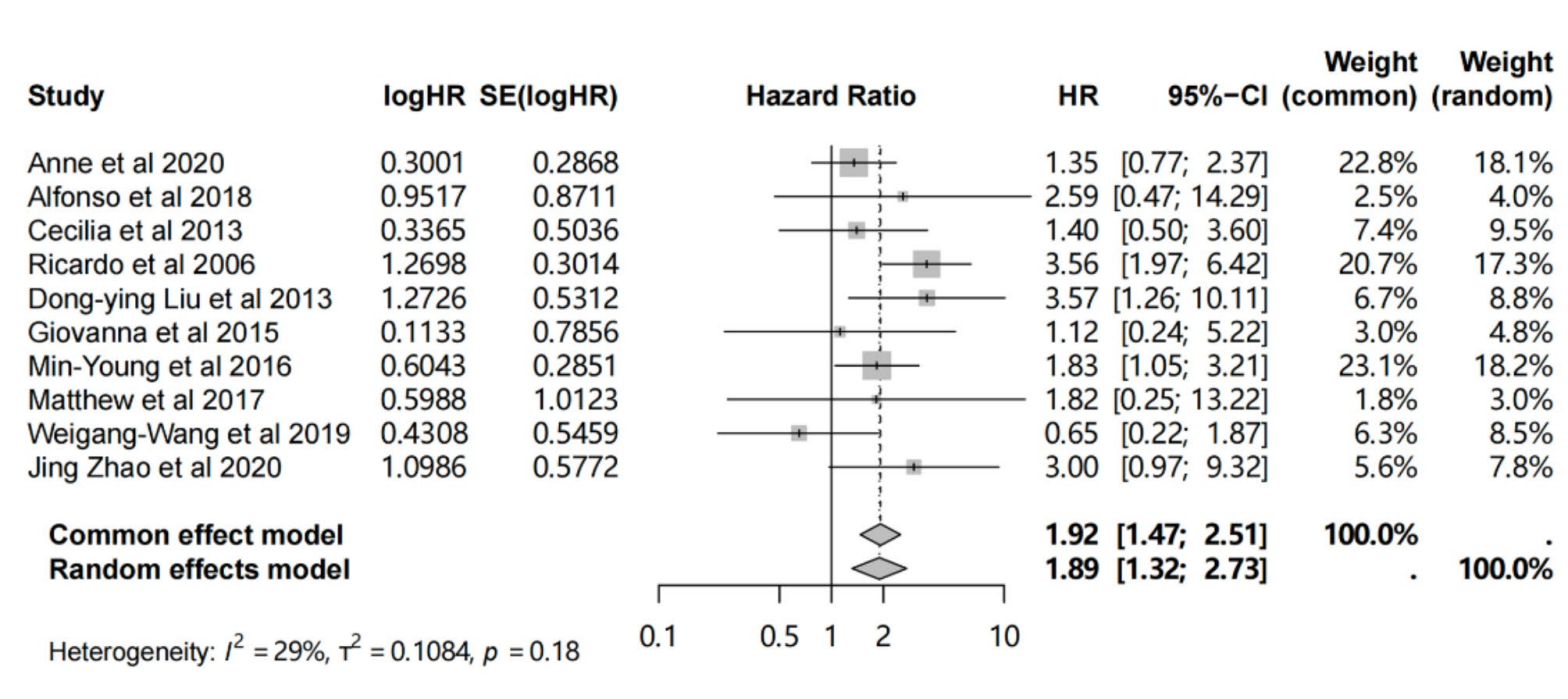
Forest plot for the correlation of HER2 + status with OS in patients with MBC.

### The prognostic role of HER2 expression in MBC

Ten studies reported a correlation between HER2 expression and OS outcomes (Table 2), using both direct HRs and indirect data extracted from survival curves. As shown in Fig. 5, the unfavorable prognostic significance of HER2-positivity was observed in the pooled analysis (HR = 1.92, 1.47–2.51) with low heterogeneity (*p* = 0.18, I^2^ = 28%). No publication bias was found in the funnel plot (*p* = 0.79) (Supplementary Fig. 2).

**Table 2:** Basic information of 10 studies which involved in analysis of the association between HER2 expression and OS outcome.

Study	PMID	Author	Year	Data source	HR	95%CI		Methods
						Lower limit	Upper limit	
1	32,108,307	Anne et al.	2020	direct	1.35	0.77	2.37	IHC + SISH
2	28,984,296	Alfonso et al.	2018	direct	2.59	0.47	14.29	PAM50
3	22,928,693	Cecilia et al.	2013	direct	1.40	0.50	3.60	IHC + SISH
4	17,001,161	Ricardo et al.	2006	indirect	3.28	1.75	6.14	IHC
5	17,001,161	Ricardo et al.	2006	indirect	3.56	1.97	6.42	FISH
6	23,751,479	Dong-ying Liu et al.	2013	indirect	3.57	1.26	10.11	IHC + FISH
7	25,948,676	Giovanna et al.	2015	indirect	1.12	0.24	5.22	IHC + FISH
8	27,100,414	Min-Young et al.	2016	direct	1.83	1.05	3.21	IHC
9	28,350,011	Matthew et al.	2017	direct	1.82	0.25	13.22	IHC + FISH
10	30,368,484	Weigang-Wang et al.	2019	indirect	0.65	0.22	1.87	NA
11	32,930,510	Jing Zhao et al.	2020	indirect	3.00	0.97	9.32	IHC + FISH

### HER2 signaling status of MBC in database

**Fig. 6:**
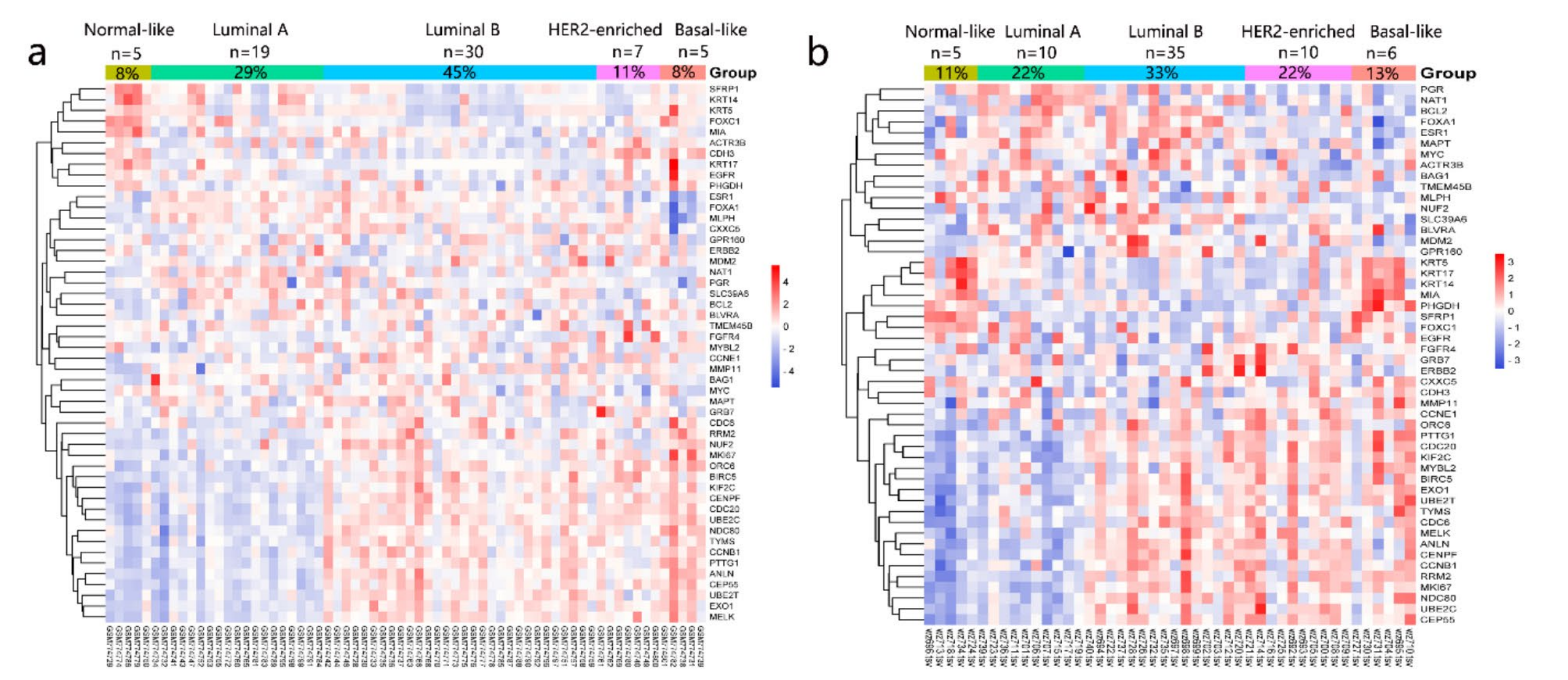
Distribution of PAM50 subtypes within GSE datasets and their 50-genes.

We extracted the transcriptional profiles of 112 MBC patients from the GEO database and identified the molecular subtype through the analysis of expression profiles of 50 genes. After excluding duplicates, 7 samples (11%) were HER2-enriched samples among 66 patients in GSE31259^65^. 10 HER2-enriched cases were found among 46 MBC (22%) in GSE104730^66^. Heatmaps of the 50 genes’ expression levels were shown in Fig. 6.

**Fig. 7:**
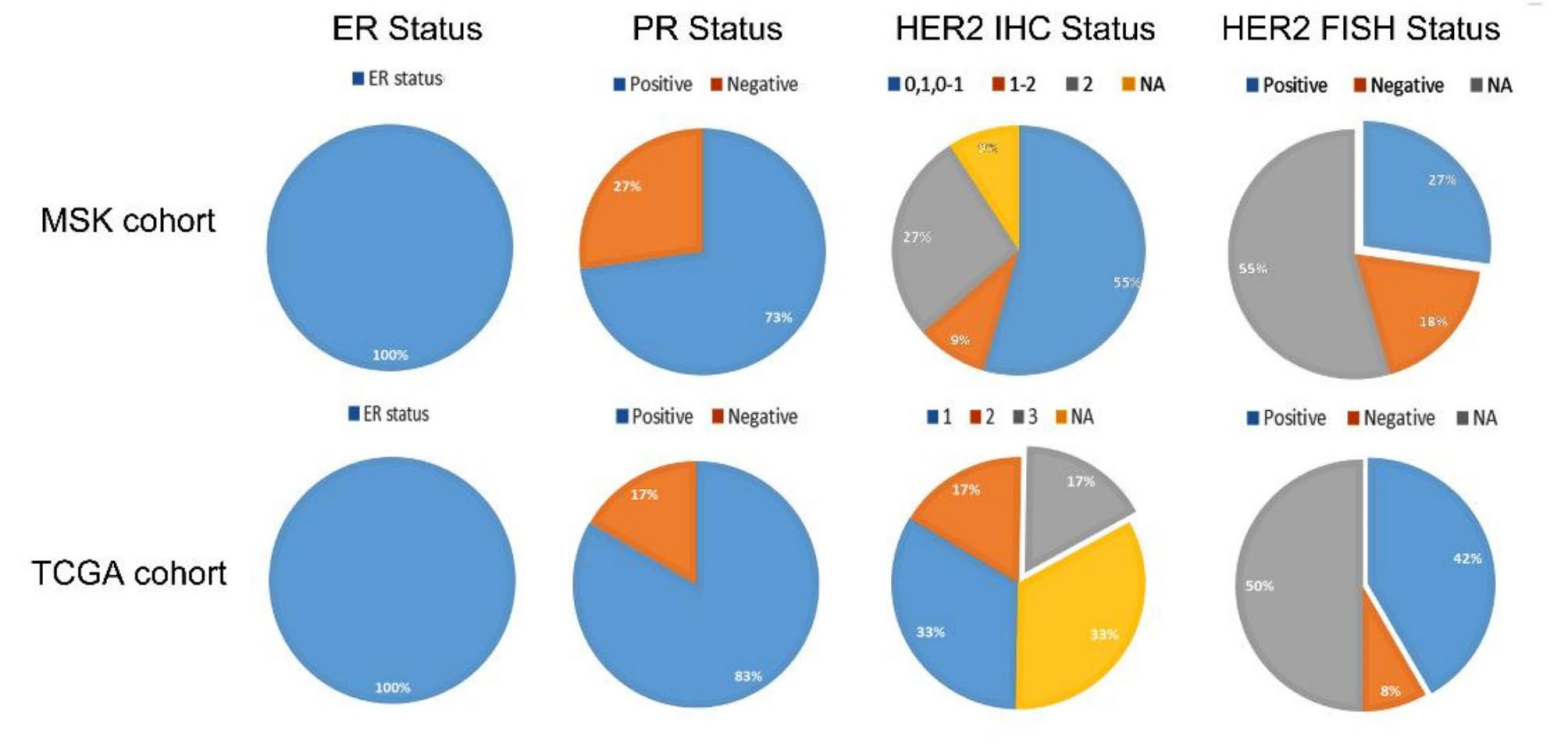
ER, PR and HER2 IHC, FISH status among MBC MSK cohort and TCGA cohort.

Additionally, 23 MBC samples were obtained from TCGA and MSK databases. In the MSK cohort, 3 patients were positive through in situ hybridizations, and HER2 2 + were observed in 3 (27%) samples (Fig. 7). In the TCGA cohort, HER2 2 + and 3 + were detected in two cases, respectively. But HER2 gene amplification was observed in five cases (42%).

**Fig. 8:**
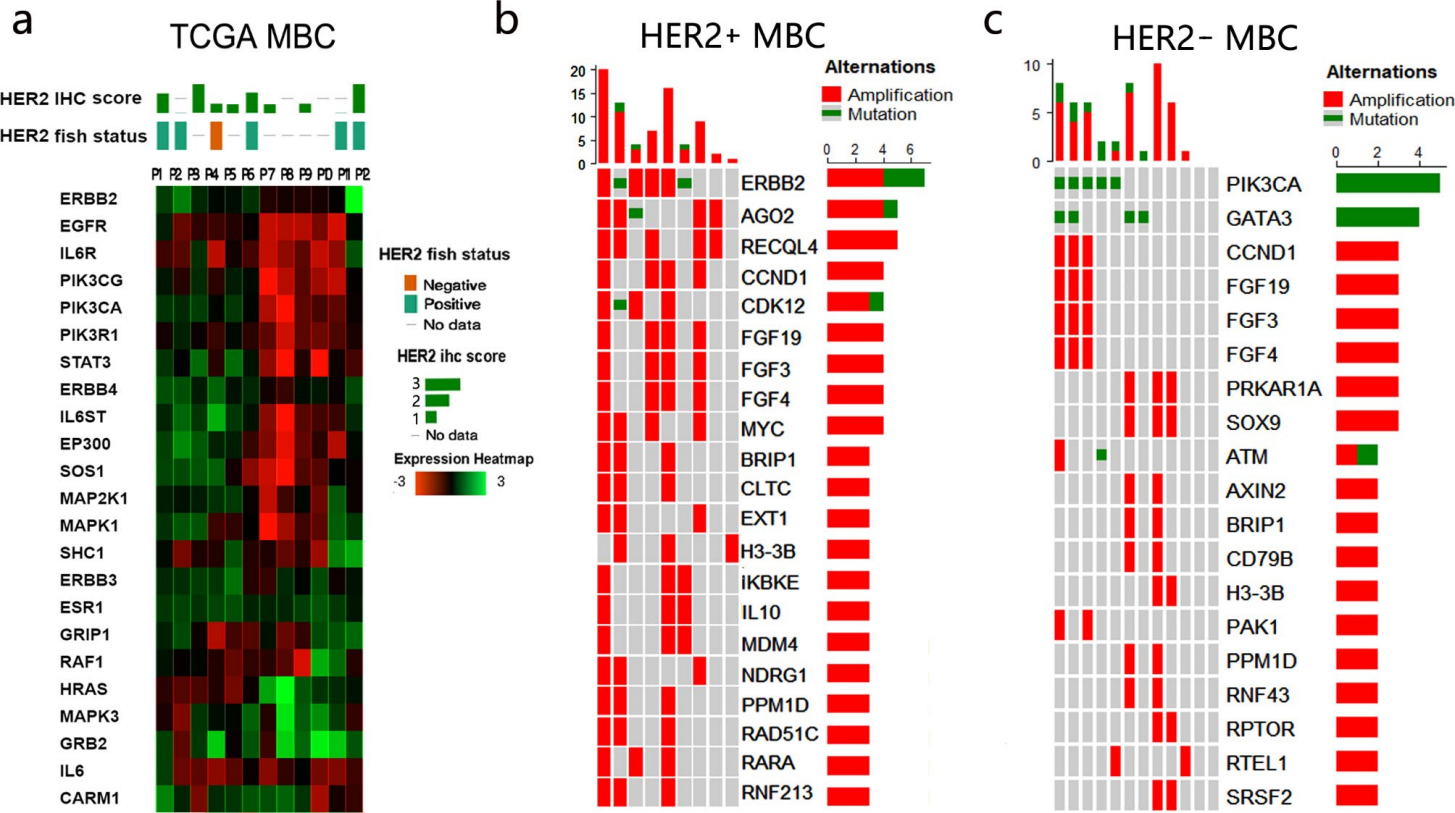
Genomic and transcriptional features of MBC patients. (a)The heatmap of genes involved in “Biocarta her2 pathway” among TCGA MBC patients, and genomic features of (b) HER2 + MBC and (c) HER2-MBC.

Using gene data from TCGA MBC patients involved in the “Biocarta HER2 Pathway”, we generated a heatmap and performed clustering to describe the activation of ERBB2 signaling transduction, as shown in Fig. 8a. We found inconsistencies between the activity of HER2 signaling and HER2 IHC status/FISH status.

### Genomic alterations related to HER2 and pathway analysis in MBC

The top 21 altered (mutated or amplified) genes in HER2 + MBC patients and HER2- MBC patients from TCGA and MSK datasets were shown in Fig. 8b and c respectively. The largest rate of ERBB mutation and amplification was 67%, followed by AGO2 and RECQL4 (56%). High frequency of genetic amplification was found in CCND1, CDK12, FGF19, FGF3, FGF4, and MYC (44%). The other 12 HER2-MBC patients had a high frequency of gene alterations in PI3KCA (42%) and GATA3 (33%), followed by PRKAR1A, SOX9, CCND1, FGF19, FGF3, and FGF4 (25%). The FGF family, CCND1, BRIP1, and PPM1D were observed in both groups with a high frequency of alterations. The altered gene lists in HER2 + FBC were displayed in Supplementary Table S2, which differed from those in HER2 + MBC and HER2- MBC.

**Fig. 9:**
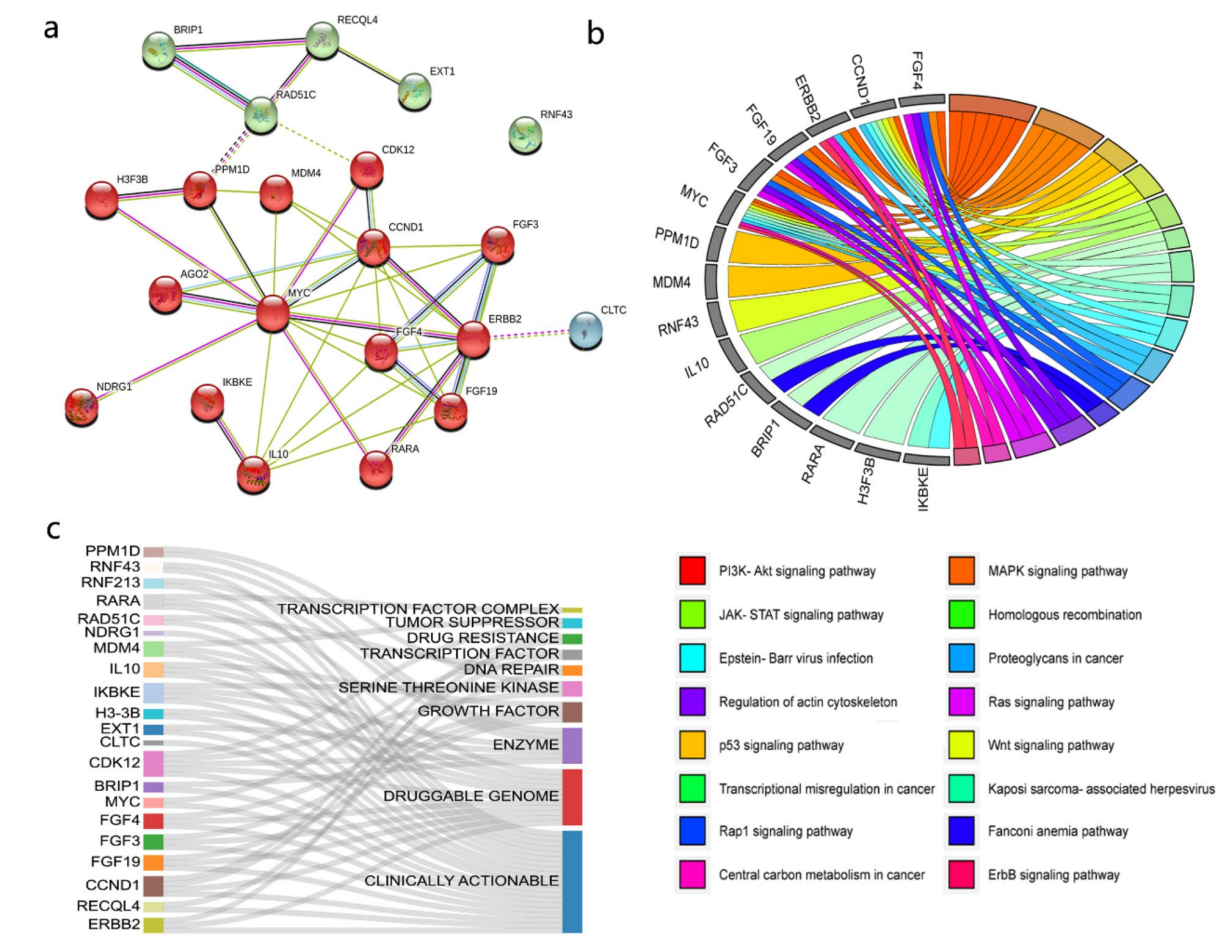
Bioinformatics analysis for genes with high frequency of alterations in HER2 + MBC. (a) the PPI network, (b) KEGG analysis and (c) drug-gene interaction analysis.

The list of genes, which are frequently mutated in HER2 + MBC was uploaded to identify the biological processes involved. The PPI network and KEGG analysis showed that genes such as FGF4, FGF19, ERBB2, FGF3, CCND1, MYC, and MDM4 (Fig. 9a) were enriched in several major signal transduction pathways, including the PI3K-Akt, MAPK, P53, and RAS signaling pathway and so on. Pathways related to the immune response (JAK-STAT) and genetic instability (homologous recombination) were enriched at the same time (Fig. 9b). We also examined the gene list in the database that processes drug-gene interactions.The majority of the genes were potential targets and clinically actionable, as shown in the Sankey diagram (Fig. 9c).

## Discussion

Current studies agree that the majority of MBC are hormone receptor positive. It is inappropriate to treat these patients as a whole, but quite difficult to categorize them. In our previous study, we identified a unique subtype of MBC: triple-positive MBC with ER+, PR + and HER2+ (TP-MBC). The prognosis of TP-MBC is worse than that of FBC, after matching all other clinical and pathological features^66^. Interestingly, we found that the ratio of TP-MBC was approximately 10% in all MBC patients, similar to that of TP-FBC. HER2 is acknowledged as one of the most successful targets for precision therapy. Thus, there is a great need to determine the rate of HER2 overexpression in MBC patients and to assess the activation of HER2 signaling, as well as the genomic features of HER2 + MBC.

First, our results revealed that the pooled percentage of HER2 + MBC patients was 10%. The prevalence was associated with the methods used to detect the status of HER2. In 31/45 studies that used IHC combined with ISH or ISH to identify ERBB2 amplification, the rate of HER2 positivity was 10%. When IHC alone was used to identify HER2 positivity, an analysis of eight studies reported a lower prevalence of HER2 positivity (7%) than the other two groups. Compared to FBC, HER2 overexpression and/or amplification appeared less frequently in MBC^17^. Our results were lower than the 14.9% reported by Mariana^17^and 17% reported by Muir^28^, and the 15% rate of HER2 amplification described by Curigliano^27^. Additional subgroup analysis revealed that race and ethnicity had an effect on HER2 overexpression status. Asia had a high positive rate of 17%. In a systematic review reported by Elahe^67^, the rate of HER2 + cases ranged from 23.3 to 81.0% in Iran, which confirmed our results.

The pooled prevalence of HER2 + MBC has become increasingly stable over the past twenty-one years. Notably, there was a fluctuation from 2001 to 2015, which might correspond to the adjustments of HER2 detection guidelines in 2007, 2013 and 2018, including the cutoff thresholds for IHC and FISH. However, there was a progressive decline from 2016 to 2022. It must be mentioned that the prevalences of HER2 + MBC extracted from each study were based on the standard at the time of publication.

The prognostic significance of HER2 in MBC is controversial^13,14,16,50^. Our analysis showed that HER2 + MBC patients had worse prognosis. Using FISH as the decision criterion for HER2 positivity rather than IHC resulted in a more significant difference in prognosis^30^. In summary, accurate determination of HER2 status is necessary.

In order to reveal the intrinsic characteristics of MBC, many studies have investigated gene expression data. The PAM50 and 21-gene RT-PCR assays are well-known tools. In PAM50, the HER2-enriched subtype refers to cancers driven primarily by HER2 pathway signaling. Their transcriptomic characteristics are similar to those of HER2 + tumors. Despite the absence of HER2 gene amplification or overexpression, this subtype has been reported to be a valid biomarker for anti-HER2 treatment^68^. Our results showed a high percentage of MBC patients with HER2 signal activation. Unfortunately, neither HER2 protein expression in tissues nor HER2 amplification was reported in these 112 MBC patients from GEO database.

We observed a lack of correlation between HER2 status by IHC/FISH and modes of HER2 downstream signals. It is possible and explicable for the HER2 gene, mRNA to be inconsistent with HER2 protein expression and the downstream pathway status^69^. In a previous study by Zelli et al., MBC was divided into two groups (cluster 1 and 2) by unsupervised clustering analysis^70^. Cluster 1 had higher expression scores for proliferation, HER2 signaling and immune responses than Cluster 2. Notably, the OS of cluster1 was worse than those of Cluster 2. However, there was no significant difference between two groups with respect to clinicopathological characteristics. Similar results were reported by Johansson et al., who found luminal M1 MBC tumors presented higher scores than luminal M2 MBC tumors for tumor invasion, proliferation, and HER2 modules. Lumina M1 MBC showed more aggressive phenotypes and worse prognosis^71^. Hence, some MBC patients with potential HER2 activation may benefit from anti-HER2 therapy, yet they never receive trastuzumab^70^.

Our genomic analysis has shown that the high-frequency altered genes differed between the HER2 + and HER2 − MBC samples. Piscuoglio et al. used whole exon sequencing to report the genomic alterations in MBC. The patients involved in their study were luminal type, and all but two were HER2+. Similar to our HER2-MBC results, PIK3CA and GATA3 were listed as the common mutated genes in their cohort^48^. The difference between the HER2 + MBC and HER2 + FBC groups may potentially be partially explained by genomic differences. In HER2 + MBC cohort of our study, genes such as MYC and CCND1 were also reported to be poor survival predictors of MBC^72^. Additionally, the copy number alteration of CLTC was suggested to be a poor prognostic factor by Moelans^73^.

On the other hand, the association between pathogenic variants and hereditary breast cancer is well established in previous studies. Most of the related genes such as BRCA1/2, PALB2, TP53, ATM, etc., are involved in DNA damage repair or act as tumor suppressors. The subgroup of hereditary breast cancer has attracted attention in recent years. We have noticed that BRIP1 has been identified as a potential germline mutation in 81 MBC cases^6^. Our research revealed that its genomic alterations were observed in both HER2 + MBC and HER2-MBC patients. ATM was was found to be changed in HER2-MBC, while the evidence of its pathogenic variants was estimated in FBC^74^. It still needs more target sequence data to illustrate the relationship between HER2 and germline mutations in MBC.

Through drug-gene interaction analysis, we illustrated that the majority of frequently altered genes in HER2 + MBC are clinically actionable. At the same time, the existence of “HER2-low” breast cancer, which exhibits a favorable response to HER2-targeted antibody-drug conjugates (ADCs) has been clarified by many studies. Further research is required on HER2-low data in men.

Our study had some limitations. First, it is better to consider the details of IHC and FISH, such as the sensitivity and specificity of antibodies in our study. Second, the heterogeneity of HER2 in tumor tissue was not analyzed in our study because of a lack of data. Finally, this study was limited by the use of indirect data and its retrospective design.

## Conclusion

Overall, our study has reduced the uncertainty about the percentage of HER2 + MBC patients and the role of HER2 expression. We demonstrated their signaling activation status using comprehensive data, contributing to a better understanding of MBC biology. There are many concerns regarding HER2 + MBC, particularly in selecting an appropriate population for anti-HER2 treatment. It is important to consider additional diagnostic tools and effective drugs.

## Electronic supplementary material

Below is the link to the electronic supplementary material.

Supplementary Material 1

## Data Availability

Data that support the findings of this study have been deposited in the related articles and in the cBioPortal, GEO database with the accession code GSE31259; GSE104730.
