# Supplementary material for: HER2 expression and pathway status in male breast cancer patients: results of an integrated analysis among 6,150 patients
Source: Sci Rep. 2025 Jan 27;15:3354. doi: 10.1038/s41598-025-86556-0 (PMC11772752; doi:10.1038/s41598-025-86556-0)
Supplement: Supplementary file 1 — Supplementary Material 1 [file 41598_2025_86556_MOESM1_ESM.docx]

HER2 expression and pathway status in male breast cancer patients: results of an integrated analysis among 6,150 patients

Boqiang Lyu^1†^, Shidi Zhao^2†^, Hui Wang^2^, Shouping Gong^1,3^, Biyuan Wang^4*^

Corresponding author: Biyuan Wang. Address: The Second Affiliated Hospital of Xi'an Jiaotong University, Xi'an, Shaanxi Province 710014, China. E-mail: wbyxjtu@163.com. ORCID: 0000-0001-6025-2929.

**Supplementary Fig S1. The funnel plot of 45 studies involved in analysis.**


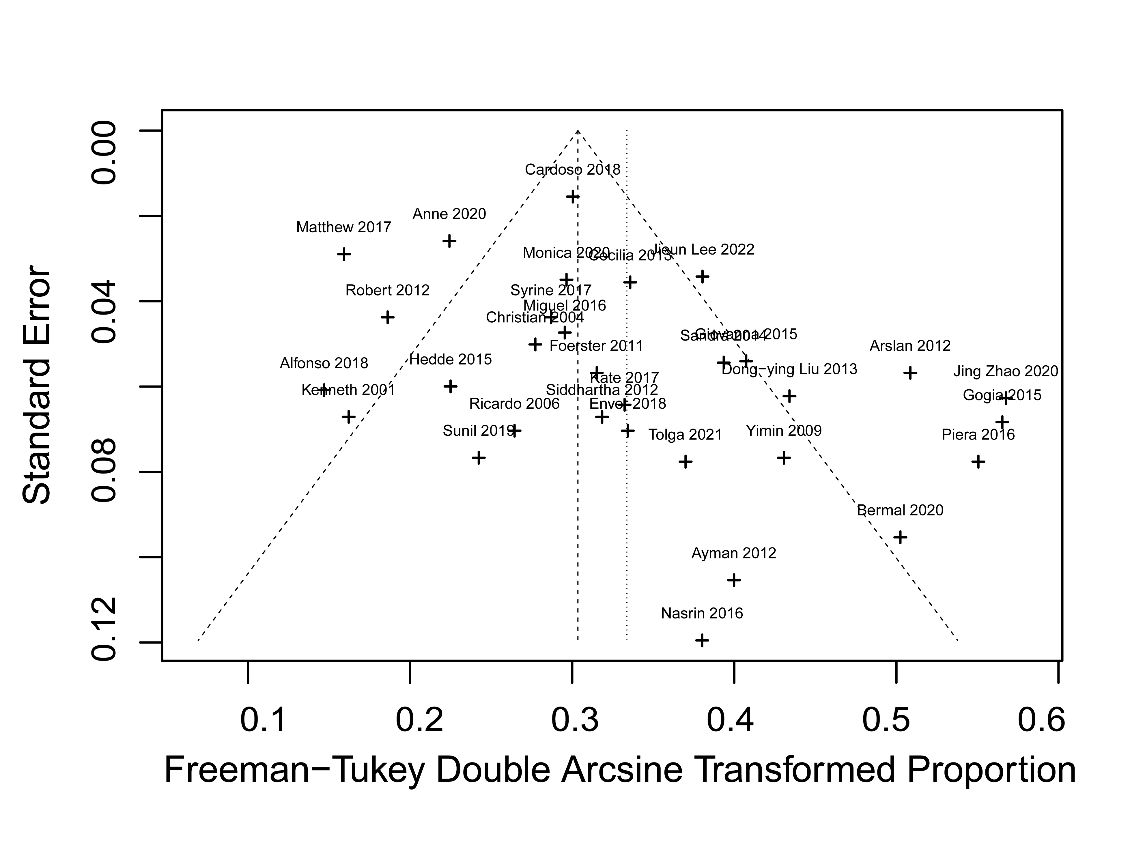


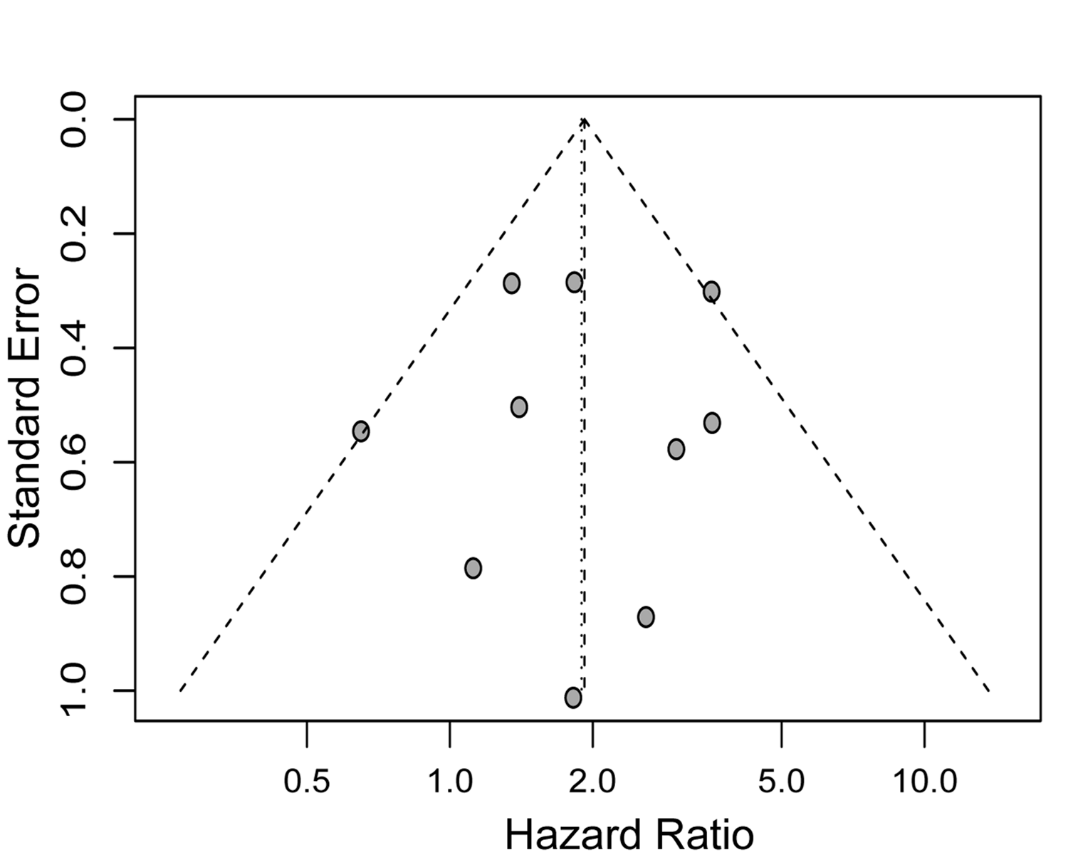
**Supplementary Fig S2. The funnel plot of 10 studies involved in survival analysis.**

| Study | Q1 | Q2 | Q3 | Q4 | Q5 | Q6 | Q7 | Q8 | Q9 | Q10 | Score |
| --- | --- | --- | --- | --- | --- | --- | --- | --- | --- | --- | --- |
| Kenneth et al.2001 | N | Y | Y | N | Y | N | Y | N | Y | Y | 6 |
| Curigliano et al.2002 | Y | Y | Y | Y | Y | N | Y | N | Y | Y | 8 |
| Muir et al.2003 | N | Y | Y | Y | Y | N | Y | Y | N | Y | 7 |
| Christian et al.2004 | Y | Y | Y | Y | Y | N | Y | N | Y | Y | 8 |
| Ricardo et al.2006 | N | Y | Y | N | N | Y | Y | Y | Y | Y | 7 |
| Kelly et al.2007 | Y | Y | Y | Y | Y | Y | Y | Y | Y | Y | 10 |
| Parveen et al.2009 | Y | Y | Y | Y | Y | Y | Y | N | Y | Y | 9 |
| Yimin et al. 2009 | N | Y | Y | Y | Y | Y | Y | N | Y | Y | 8 |
| Foerster et al.2011 | Y | Y | Y | Y | Y | Y | Y | Y | Y | Y | 10 |
| Arslan et al.2012 | N | Y | Y | Y | Y | Y | Y | Y | Y | Y | 9 |
| Ayman et al.2012 | N | Y | Y | Y | Y | Y | Y | N | Y | Y | 8 |
| Robert et al.2012 | N | Y | Y | Y | Y | Y | Y | N | Y | Y | 8 |
| Abeer et al.2012 | N | Y | Y | N | N | Y | Y | Y | Y | Y | 7 |
| Siddhartha et al.2012 | Y | Y | Y | Y | Y | Y | Y | Y | Y | Y | 10 |
| Nicla et al.2013 | Y | Y | Y | Y | Y | Y | Y | Y | Y | Y | 10 |
| Cecilia et al.2013 | Y | Y | Y | Y | Y | Y | Y | Y | Y | Y | 10 |
| Mariana et al.2013 | Y | Y | Y | Y | Y | Y | Y | Y | Y | Y | 10 |
| Dong-ying Liu et al.2013 | N | Y | Y | Y | Y | Y | Y | Y | Y | Y | 9 |
| Sandro et al.2014 | Y | Y | Y | Y | Y | Y | Y | Y | Y | Y | 10 |
| Gogia et al.2015 | N | Y | Y | Y | Y | Y | Y | Y | Y | Y | 9 |
| Giovanna et al.2015 | N | Y | Y | Y | Y | Y | Y | Y | Y | Y | 9 |
| Hedde et al.2015 | N | Y | Y | Y | Y | N | Y | N | Y | Y | 7 |
| Min-Young et al.2016 | Y | Y | Y | Y | Y | Y | Y | Y | Y | Y | 10 |
| Nasrin et al.2016 | N | Y | Y | Y | Y | Y | Y | Y | Y | Y | 9 |
| Piera et al.2016 | Y | Y | Y | Y | Y | Y | Y | Y | Y | Y | 10 |
| Miguel et al.2016 | N | Y | Y | Y | Y | Y | Y | Y | Y | Y | 9 |
| Salvatore et al.2016 | Y | Y | Y | Y | Y | Y | N | N | Y | Y | 8 |
| Kate et al.2017 | N | Y | Y | Y | Y | Y | Y | Y | Y | Y | 9 |
| Matthew et al.2017 | N | Y | Y | Y | Y | Y | Y | Y | Y | Y | 9 |
| Syrine et al.2017 | N | Y | Y | Y | Y | Y | Y | Y | Y | Y | 9 |
| Enver et al.2018 | Y | Y | Y | Y | Y | Y | Y | Y | Y | Y | 10 |
| Alfonso et al.2018 | N | Y | Y | Y | Y | Y | Y | Y | Y | Y | 9 |
| Quirine et al.2018 | N | Y | Y | Y | Y | Y | N | Y | Y | Y | 8 |
| Cardoso et al.2018 | Y | Y | Y | Y | Y | Y | Y | Y | Y | Y | 10 |
| Weigang-Wang et al.2019 | N | Y | Y | Y | Y | Y | Y | Y | Y | Y | 9 |
| Sunil et al.2019 | N | Y | Y | Y | Y | Y | N | Y | Y | Y | 8 |
| Piera et al.2019 | N | Y | Y | Y | Y | Y | N | N | Y | Y | 7 |
| Jing Zhao et al.2020 | Y | Y | Y | Y | Y | Y | Y | Y | Y | Y | 10 |
| Bermal et al.2020 | Y | Y | Y | Y | Y | Y | N | Y | Y | Y | 9 |
| Monica et al.2020 | Y | Y | Y | Y | Y | Y | Y | Y | Y | Y | 10 |
| Anne et al.2020 | Y | Y | Y | Y | Y | Y | Y | Y | Y | Y | 10 |
| Guo-Sang et al.2021 | Y | Y | Y | Y | Y | Y | Y | Y | Y | Y | 10 |
| Tolga et al.2021 | N | Y | Y | Y | Y | Y | Y | Y | Y | Y | 9 |
| Marek et al.2021 | N | Y | Y | Y | Y | Y | N | N | Y | Y | 7 |
| Jieun Lee et al.2022 | Y | Y | Y | Y | Y | Y | Y | Y | Y | Y | 10 |

**Supplementary Table S1. JBI score of 45 studies included in analysis.**

**Supplementary Table S2.** **The genomic altered gene lists in HER2+ FBC from MSK and TCGA database.**

| Mutated Gene | MSK-Cohort | | TCGA-Cohort | | Overall | | Gene Amplifi  -cation | MSK-Cohort | | TCGA-Cohort | | | Overall |
| --- | --- | --- | --- | --- | --- | --- | --- | --- | --- | --- | --- | --- | --- |
|  | Mutated Samples  n=199 | % | Mutated Samples  n=159 | % | | % |  | Amp Samples  n=199 | % | | Amp Samples  n=175 | % | % |
| TP53 | 117 | 58.80 | 61 | 38.40 | | 49.70 | ERBB2 | 165 | 82.90 | | 95 | 54.30 | 69.52 |
| PIK3CA | 64 | 32.20 | 47 | 29.60 | | 31.00 | CDK12 | 113 | 56.80 | | 87 | 49.70 | 53.48 |
| CDH1 | 21 | 10.60 | 10 | 6.30 | | 8.60 | RARA | 40 | 20.10 | | 44 | 25.10 | 22.46 |
| GATA3 | 17 | 8.50 | 11 | 6.90 | | 7.80 | CCND1 | 34 | 17.10 | | 39 | 22.30 | 19.52 |
| KMT2C | 11 | 5.50 | 10 | 6.30 | | 5.80 | SPOP | 31 | 15.60 | | 41 | 23.40 | 19.25 |
| NF1 | 15 | 7.50 | 5 | 3.10 | | 5.50 | FGF19 | 31 | 15.60 | | 39 | 22.30 | 18.72 |
| KMT2D | 11 | 5.50 | 6 | 3.80 | | 4.70 | MYC | 18 | 9.00 | | 52 | 29.70 | 18.72 |
| ERBB2 | 11 | 5.50 | 5 | 3.10 | | 4.40 | FGF4 | 28 | 14.10 | | 39 | 22.30 | 17.91 |
| RUNX1 | 10 | 5.00 | 6 | 3.80 | | 4.40 | FGF3 | 27 | 13.60 | | 38 | 21.70 | 17.38 |
| ERBB3 | 8 | 4.00 | 8 | 5.00 | | 4.40 | RAD21 | 11 | 8.10 | | 47 | 26.90 | 15.51 |
| ATM | 8 | 4.00 | 7 | 4.40 | | 4.10 | RAD51C | 21 | 10.60 | | 33 | 18.90 | 14.44 |
| PTEN | 9 | 4.50 | 5 | 3.10 | | 3.90 | PPM1D | 13 | 9.60 | | 41 | 23.40 | 14.44 |
| ZFHX3 | 6 | 4.40 | 6 | 3.80 | | 3.30 | RNF43 | 12 | 6.00 | | 36 | 20.60 | 12.83 |
| PTPRD | 7 | 3.50 | 5 | 3.10 | | 3.30 | HOXB13 | 14 | 10.30 | | 32 | 18.30 | 12.30 |
| ARID1A | 6 | 3.00 | 6 | 3.80 | | 3.30 | PRKAR1A | 16 | 8.00 | | 30 | 17.10 | 12.30 |
| TBX3 | 10 | 5.00 | 1 | 0.60 | | 3.00 | CD79B | 14 | 7.00 | | 32 | 18.30 | 12.30 |
| FOXA1 | 7 | 3.50 | 4 | 2.50 | | 3.00 | BRIP1 | 10 | 5.00 | | 33 | 18.90 | 11.50 |
| FLT4 | 8 | 4.00 | 2 | 1.30 | | 2.70 | NBN | 9 | 4.50 | | 31 | 17.70 | 10.70 |
| ARID2 | 7 | 3.50 | 3 | 1.90 | | 2.70 | MCL1 | 9 | 4.50 | | 31 | 17.70 | 10.70 |
| PRDM1 | 6 | 3.00 | 4 | 2.50 | | 2.70 | FGFR1 | 15 | 7.50 | | 22 | 12.60 | 9.89 |
